# Total tumour diameter is superior to unifocal diameter as a predictor of papillary thyroid microcarcinoma prognosis

**DOI:** 10.1038/s41598-017-02165-6

**Published:** 2017-05-12

**Authors:** Chunping Liu, Shuntao Wang, Wen Zeng, Yawen Guo, Zeming Liu, Tao Huang

**Affiliations:** 1 Department of Breast and Thyroid Surgery, Union Hospital, Tongji Medical College, Huazhong University of Science and Technology, Wuhan, People’s Republic of China; 2 Department of Ophthalmology, Zhongnan Hospital, Wuhan University, Wuhan, Hubei China

## Abstract

The current American Joint Committee (AJCC) on Cancer TNM classification does not describe the treatment of multifocal papillary thyroid microcarcinomas (PTMCs) with a total tumour diameter (TTD) >1 cm. Herein, we investigated this PTMC subgroup in terms of extrathyroidal extension (ETE), local infiltration, central lymph node metastasis (LNM), and prognosis. Consecutive patients (n = 1102) were identified and the proportions of LNM, ETE, and local infiltration were similar between PTCs with a unifocal tumour diameter >1 cm and ≤2 cm and PTMCs with a multifocal TTD >1 cm and ≤2 cm. The proportions of LNM, ETE, and local infiltration were also similar between PTMCs with a unifocal diameter ≤1 cm *vs*. multifocal TTD ≤1 cm. However, when comparing PTMCs with a unifocal diameter ≤1 cm *vs*. multifocal TTD >1 cm, significant differences were observed. In the Kaplan-Meier analysis, significant differences were observed between PTMCs with a unifocal diameter ≤1 cm *vs*. multifocal TTD >1 cm and multifocal TTD ≤1 cm *vs*. multifocal TTD >1 cm. Accordingly, TTD may represent a more accurate criterion for tumour size of PTCs and should be considered in the revised AJCC staging system.

## Introduction

According to the World Health Organization classification system, papillary thyroid microcarcinoma (PTMC) is defined as thyroid cancer measuring less than or equal to 1.0 cm in its greatest dimension^1^. PTMCs are diagnosed with increasing frequency. The proportion of PTMCs among all PTCs increased from 18.4% between 1983 and 1987 to 43.1% between 1998 and 2001 in France^2^, and similar results have been reported from other countries such as the United States and China^3–5^.

The 6^th^ edition of the American Joint committee on Cancer (AJCC) tumour, node, metastasis (TNM) classification system for differentiated thyroid cancer defines T1a tumours as those with a tumour diameter ≤1 cm (PTMC) without extrathyroidal extension (ETE), and this subgroup of patients are recommended to undergo lobectomy. However, the AJCC classification system, along with the guidelines recommended by the American Thyroid Association, defines the tumour size according to the traditional intraglandular maximal tumour diameter, and whether the subgroup of patients with multifocal PTMC and a total tumour diameter (TTD) >1 cm shares the same features and prognosis as those with traditional PTMCs remain unclear.

Hence, in the present study, we aimed to demonstrate whether the TTD should be used as a more accurate criterion for tumour size of papillary thyroid carcinomas (PTCs) and should be added as an additional prognostic factor in the AJCC classification system.

## Results

Overall, the postoperative follow-up period ranged between 18–148 months, with a median follow-up of 61.0 months. Clinicopathological characteristics of the papillary thyroid microcarcinoma patients (n = 1102) and papillary thyroid carcinoma (1 < unifocal with diameter ≤ 2 cm) (n = 210) were present in Table 1. Among the 390 multifocal PTMC cases, 32.6% (n = 127) and 67.4% (n = 263) were unilateral and bilateral, respectively. A total of 28.3% cases present Hashimoto, while 71.7% (n = 790) did not. We used the TTD of each specimen as a new parameter for the multifocal cases. The LNM, ETE, local infiltration, and multifocal or unifocal diseases were compared in subgroups according to the tumour size. The results are displayed in Tables 2–4.

**Table 1:** Basic clinicopathological characteristics of the papillary thyroid microcarcinoma patients (n = 1102) and papillary thyroid carcinoma (1 < unifocal with diameter ≤ 2 cm) (n = 210).

Characteristic	PTMC	PTC (1 < unifocal with diameter ≤ 2 cm)
	n = 1102	n = 210
Age (years)		
<45	469 (42.6%)	92 (43.8%)
≥45	633 (57.4%)	118 (56.2%)
Sex		
Female	904 (82.0%)	161 (76.7%)
Male	198 (18.0%)	49 (23.3%)
Maximal tumor size (cm)		
≤0.5	573 (52.0%)	
>0.5	529 (48.0%)	
Total tumor size (cm)		
≤1	923 (83.8%)	
>1	179 (16.2%)	
multifocality		
Absent	712 (64.6%)	
Present	390 (35.4%)	
Lymph node metastasis		
Absent	805 (73.0%)	80 (38.1%)
Present	297 (27.0%)	130 (61.9%)
Extrathyroidal invasion		
Present	288 (26.1%)	101 (48.1%)
Absent	814 (73.9%)	109 (51.9%)
Local infiltration		
Present	41 (3.7%)	178 (84.8%)
Absent	1061 (96.3%)	32 (15.2%)
Subtype		
Classic	1020 (92.6%)	
Follicular	82 (7.4%)	

PTMC: papillary thyroid microcarcinoma. PTC: papillary thyroid carcinoma.

**Table 2:** Differences in lymph node metastasis between the different tumour size groups.

Characteristic	Total	LNM negative n (%)	LNM positive n (%)	p
**PTC** ***vs*** **PTMC**				
Group A	210	130 (61.9)	80 (38.1)	0.522
Group B	85	56 (65.9)	29 (34.1)	
**PTMC**				
Group C	712	558 (78.4)	154 (21.6)	0.286
Group D	211	158 (74.9)	53 (25.1)	
Group C	712	558 (78.4)	154 (21.6)	<0.001
Group E	179	89 (49.7)	90 (50.3)	
Group D	211	158 (74.9)	53 (25.1)	<0.001
Group E	179	89 (49.7)	90 (50.3)	

Group A: PTC, unifocal with diameter >1 cm and ≤2 cm; Group B: PTMC multifocal with total tumor diameter (TTD) >1 cm and ≤2 cm; Group C: PTMC unifocal with diameter ≤1 cm; Group D: PTMC multifocal with TTD ≤1 cm; Group E: PTMC multifocal with TTD >1 cm.

**Table 3:** Differences in lymph node metastasis between the different extrathyroid extension groups.

Characteristic	Total	ETE negative n (%)	ETE positive n (%)	p
**PTC** ***vs*** **PTMC**				
Group A	210	101 (48.1)	109 (51.9)	0.838
Group B	85	42 (49.4)	43 (50.9)	
**PTMC**				
Group C	712	530 (74.4)	182 (25.6)	0.068
Group D	211	170 (80.6)	41 (19.4)	
Group C	712	530 (74.4)	182 (25.6)	0.005
Group E	179	115 (64.2)	64 (35.8)	
Group D	211	170 (80.6)	41 (19.4)	<0.001
Group E	179	115 (64.2)	64 (35.8)	

ETE: extrathyroidal extension; Group A: PTC, unifocal with diameter >1 cm and ≤2 cm; Group B: PTMC multifocal with total tumor diameter (TTD) >1 cm and ≤2 cm; Group C: PTMC unifocal with diameter ≤1 cm; Group D: PTMC multifocal with TTD ≤ 1 cm; Group E: PTMC multifocal with TTD >1 cm.

**Table 4:** Differences in lymph node metastasis between the different local infiltration groups.

Characteristic	Total	Local infiltration negative n (%)	Local infiltration positive n (%)	p
**PTC** ***vs*** **PTMC**				
Group A	210	178 (84.8)	32 (15.2)	0.439
Group B	85	75 (88.2)	10 (11.8)	
**PTMC**				
Group C	712	688 (96.6)	24 (3.4)	0.970
Group D	211	204 (96.7)	7 (3.3)	
Group C	712	685 (96.2)	21 (3.8)	0.008
Group E	179	166 (92.7)	13 (7.3)	
Group D	211	204 (96.7)	7 (3.3)	0.078
Group E	179	166 (92.7)	13 (7.3)	

Group A: PTC, unifocal with diameter >1 cm and ≤2 cm; Group B: PTMC multifocal with total tumor diameter (TTD) >1 cm and ≤2 cm; Group C: PTMC unifocal with diameter ≤1 cm; Group D: PTMC multifocal with TTD≤1 cm; Group E: PTMC multifocal with TTD >1 cm.

LNM occurred in 38.1% of PTCs with a unifocal tumour diameter of >1 cm and ≤2 cm (Group A) and in 34.1% of PTMCs with a multifocal TTD >1 cm and ≤2 cm (Group B) (p = 0.522). For PTMCs, LNM occurred in 21.6% and 25.1% of patients with a unifocal diameter ≤1 cm (Group C) and a multifocal TTD ≤ 1 cm (Group D), respectively (p = 0.286). However, when comparing PTMCs with a unifocal diameter ≤1 cm (Group C) *vs*. a multifocal TTD >1 cm (Group E), the proportions of cases with LNM significantly differed, at 21.6% and 50.3%, respectively (p < 0.001). In addition, comparison was also carried out between cases with a multifocal TTD ≤ 1 cm (Group D) *vs*. multifocal TTD >1 cm (Group E), which revealed significant differences in LNM (25.1% *vs*. 50.3%, p < 0.001) (Table 2).

ETE occurred in 51.9% of PTCs with a unifocal tumour diameter of >1 cm and ≤2 cm (Group A) and in 50.9% of PTMCs with a multifocal TTD of >1 cm and ≤2 cm (Group B) (p = 0.838). For PTMCs, ETE occurred in 25.6% of patients with a unifocal diameter ≤1 cm (Group C) and 19.4% of patients with a multifocal TTD ≤ 1 cm (Group D) (p = 0.068). However, when comparing PTMCs with a unifocal diameter ≤1 cm (Group C) to those with a multifocal TTD >1 cm (Group E), the ETE proportions significantly differed, at 25.6% and 35.8%, respectively (p = 0.005). In addition, significant differences were also observed between cases of multifocal TTD ≤ 1 cm (Group D) *vs*. multifocal TTD >1 cm (Group E) (19.4% *vs*. 35.8%, p < 0.001) (Table 3).

Local infiltration occurred in 15.2% of PTCs with a unifocal tumour diameter ≫1 cm and ≤2 cm (Group A) and in 11.8% of PTMCs with a multifocal TTD of ≫1 cm and ≤2 cm (Group B) (p = 0.439). For PTMCs, local infiltration occurred in 3.4% and 3.3% of patients with a unifocal diameter ≤1 cm (Group C) and a multifocal TTD ≤ 1 cm (Group D) (p = 0.970). However, when comparing PTMCs with a unifocal diameter ≤1 cm (Group C) *vs*. a multifocal TTD >1 cm (Group E), the proportions of cases with local infiltration were 3.8% and 7.3%, respectively, which showed a statistically significant difference (p = 0.008). In addition, comparison was also carried out between cases of multifocal TTD ≤ 1 cm (Group D) *vs*. multifocal TTD >1 cm (Group E), although no significant differences were found (3.3% *vs*. 7.3%, p = 0.078) (Table 4).

**Figure 1:**
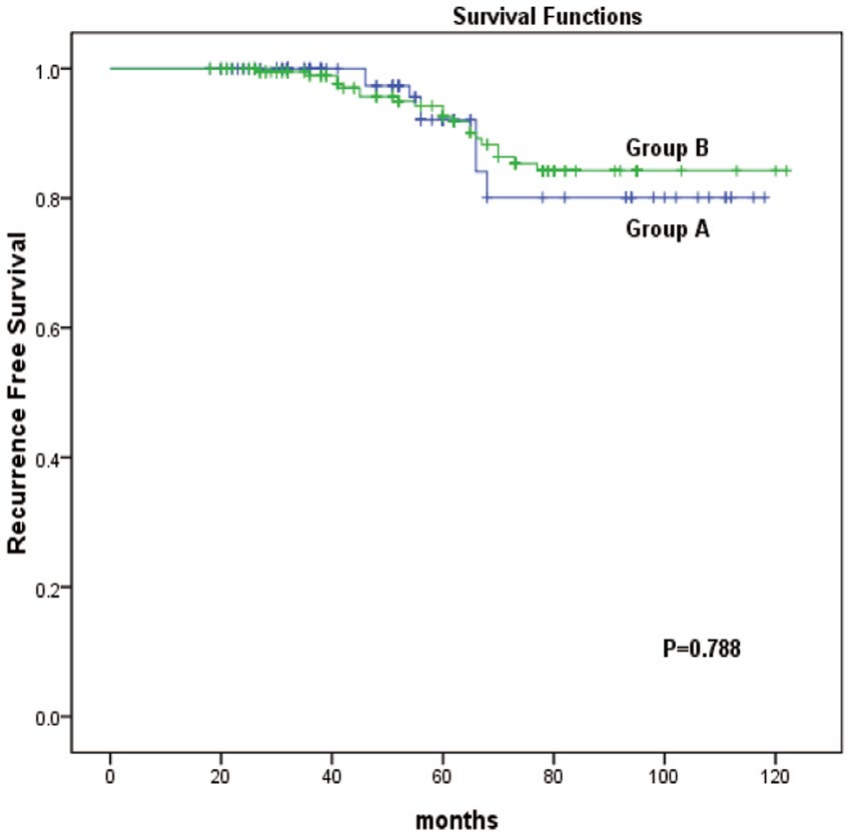
Kaplan-Meier analysis of recurrence-free survival of unifocal papillary thyroid cancer with a diameter >1 cm and ≤2 cm (group A) and multifocal papillary thyroid microcarcinoma with a total tumour diameter (TTD) >1 cm and ≤2 cm (group B).

**Figure 2:**
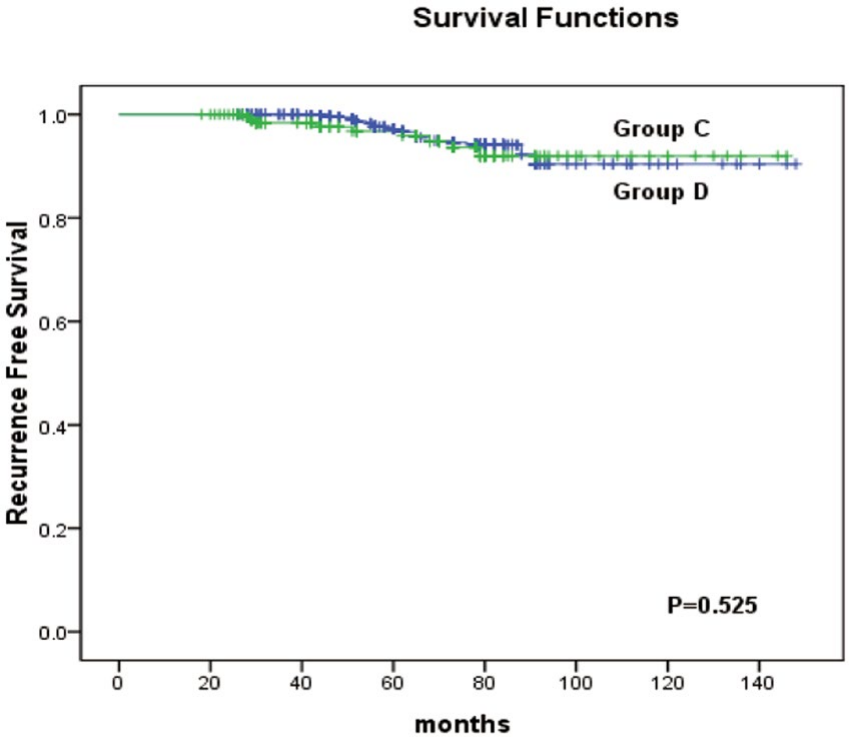
Kaplan-Meier analysis of recurrence-free survival of unifocal papillary thyroid cancer with a diameter of ≤1 cm (group C) and multifocal papillary thyroid microcarcinoma with a total tumour diameter (TTD) of ≤1 cm (group D).

**Figure 3:**
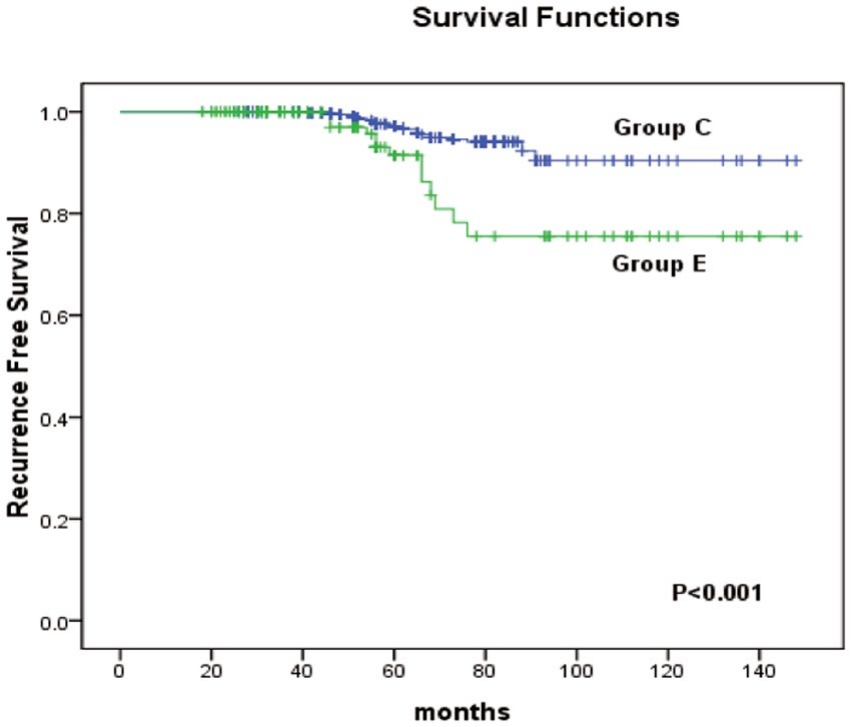
Kaplan-Meier analysis of recurrence-free survival of unifocal papillary thyroid cancer with a diameter of ≤1 cm (group C) and multifocal papillary thyroid microcarcinoma with a total tumour diameter (TTD) of >1 cm (group E).

**Figure 4:**
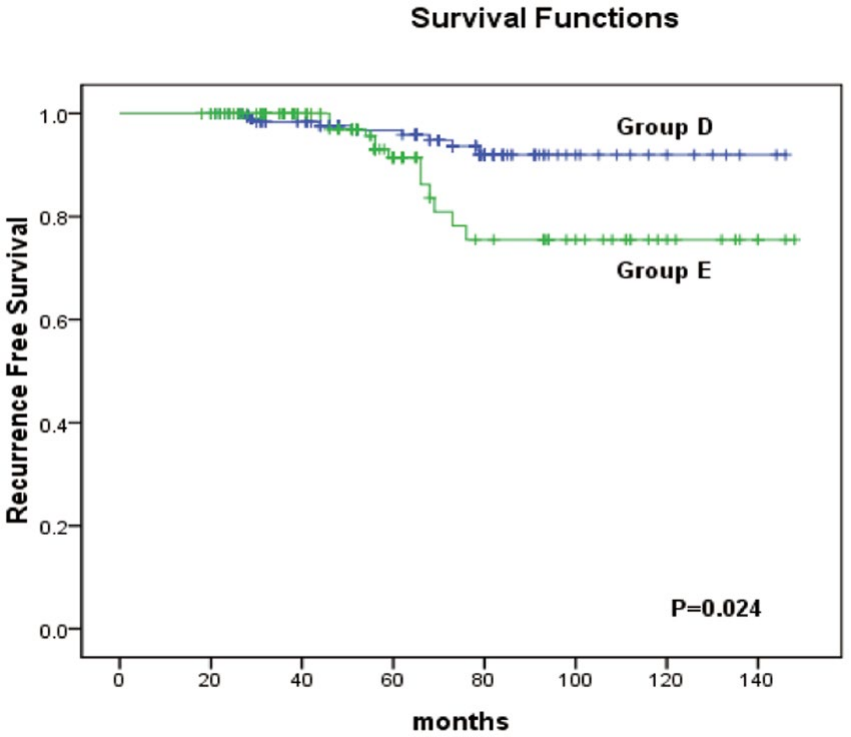
Kaplan-Meier analysis of recurrence-free survival of multifocal papillary thyroid microcarcinoma with a total tumour diameter (TTD) of ≤1 cm (group D) and multifocal papillary thyroid microcarcinoma with a total tumour diameter (TTD) of >1 cm (group E).

Finally, we examined the relationships between tumour size and the recurrence-free survival (RFS) of the patients. Kaplan-Meier analysis revealed that there was no difference in the RFS between patients with PTCs with a unifocal tumour diameter of ≫1 cm and ≤2 cm (Group A) compared to those with PTMCs with a multifocal TTD of ≫1 cm and ≤2 cm (Group B) (p = 0.788) (Fig. 1). For PTMCs, the RFS did not significantly differ between patients with a unifocal diameter ≤1 cm (Group C) and a multifocal TTD ≤ 1 cm (Group D) (p = 0.525) (Fig. 2). However, when comparing PTMCs with a unifocal diameter ≤1 cm (Group C) to those with a multifocal TTD >1 cm (Group E), a statistically significant difference was found (p < 0.001) (Fig. 3). In addition, a significant difference was also observed between cases of multifocal TTD ≤ 1 cm (Group D) and multifocal TTD >1 cm (Group E) (p = 0.024) (Fig. 4).

## Discussion

PTC is the most common malignant neoplasm originating from the thyroid gland and generally has an excellent prognosis^6^. PTMC is being diagnosed with increasing frequency worldwide and now constitutes nearly half of all cases of PTC^2, 7, 8^. The primary operative interventions for PTCs with tumour size >1 cm and PTMC differ^6^, and, because of this, clinicians are faced with the challenge of detecting and managing multifocal PTMC efficiently.

The TNM staging system is based on the extent of the primary tumour (T), the presence of regional lymph node metastasis (N), and the identification of distant metastasis (M)^6^. For PTC, including PTMC, the current AJCC TNM staging system defines the tumour size as the greatest dimension of the largest solitary tumour in cases of multifocal tumours^6^. Multifocality has been reported to occur in 30–40% of PTMCs^9–11^. A close relationship between larger tumour size and worse outcome, both in terms of recurrence and survival, has been well established and is generally accepted^6, 12–14^. However, it remains unclear whether multifocal PTMCs with a TTD >1 cm are associated with worse outcomes compared to those with a TTD ≤ 1 cm.

A large Surveillance, Epidemiology, and End Results database study of 52,173 patients by Bilimoria *et al*.^15^ concluded that PTCs >1 cm were associated with a worse prognosis, and more extensive thyroid surgery was thus required for these patients. This finding impacted the decision to revise the AJCC classification system to subdivide the T1 category for intraglandular tumours ≤2 cm into tumours ≤1 cm and >1 cm and ≤2 cm in size. Similarly, the various other systems classifying tumours into high- and low-risk groups include tumour size as a one of the prognostic grouping factors; however, the size thresholds range widely^15–17^.

In our results, the risks of LNM, ETE, and local infiltration, as well as the RFS, did not significantly differ between PTCs with a unifocal diameter >1 cm and ≤2 cm and PTMCs with a multifocal TTD >1 cm and ≤2 cm or between PTMCs with a unifocal diameter ≤1 cm and multifocal TTD ≤ 1 cm. However, the risks of LNM, ETE, and infiltration and the RFS were significantly different between PTMCs with a unifocal diameter ≤1 cm and multifocal TTD >1 cm and between multifocal PTMCs with a TTD of ≤1 cm and >1 cm, except for local infiltration. The latter finding may be due to the included PTMCs mostly being early-stage tumours and rarely presenting local infiltration.

The main argument for treatment such as prophylactic central lymph node metastasis or radioiodine ablation in PTC and PTMC patients is based on maximum clearance of the tumour, which could result in a decreased rate of recurrence, improved survival, and avoidance of reoperations in the central neck compartment^18–21^. Multifocality has been reported to be a negative prognostic factor for recurrence and is considered an important risk factor in many publications^22–26^. Further, it is still unclear whether PTMC should be considered a separate clinical entity that requires different management compared to conventional PTC^9^; therefore, the size of all foci should be considered when estimating the tumour mass for multifocal PTMC.

In our previous study, we found that multifocal PTMCs with a TTD >1 cm were associated with a similar risk of LNM as PTCs^1^. In this study, we further demonstrated that multifocal PTMCs with a TTD >1 cm were also associated with similar risks of high-risk clinicopathological features (LNM, ETE) and similar RFS as PTC. Overall, the above findings indicate that, in multifocal microcarcinomas, the diameter of the largest lesion cannot be directly used as an efficient tool to predict the risks of LNM, ETE, and local infiltration, as well as the prognosis.

Our data have some limitations. First, this study was not a randomised case-control trial; however, we consider our findings to reflect the total PTMC patient population surgically treated at one institutions. In addition, our cohort of patients had a relatively short median follow-up of 61 months. A longer follow-up is required to make any definitive conclusions regarding the effect of the TTD of multifocal PTMCs on the RFS.

## Conclusion

The TTD may represent a more accurate criterion for tumour size of PTMCs. Our results suggest that the classification of tumour size according to the dimension of the largest focus for multifocal PTMCs, as advocated by the AJCC and currently used as a treatment recommendation by the American Thyroid Association, needs to be revisited.

## Methods

### Study Population

We conducted a retrospective review of consecutive patients who underwent total-thyroidectomy plus prophylactic central lymph node dissection in our medical centre (Union Hospital) between January 2003 and November 2014. Data were obtained from our clinical database, all methods were performed in accordance with the relevant guidelines and regulations, the study protocol was approved by our institutional review boards (Union Hospital) and the file number: 20131001PTMC. Written informed consent from all participants were obtained.

A total of 1102 patients with PTMC were included in the analysis. Additionally, 210 patients with unifocal PTC with a diameter of more than 1 cm but less than or equal to 2 cm were also included for comparisons with the PTMC cases. Demographic information, surgical details, histopathologic details, and postoperative use of radioactive iodine were recorded. Postoperative thyroid-stimulating hormone suppression was practiced based on the recurrence risk for all patients. The clinicopathological parameters, including radioactive iodine remnant ablation status were also recorded and compared. The subgroups were organized: Group A: PTC, unifocal with diameter >1 cm and ≤2 cm (n = 210); Group B: PTMC multifocal with total tumor diameter (TTD) >1 cm and ≤2 cm (n = 85); Group C: PTMC unifocal with diameter ≤1 cm (n = 712); Group D: PTMC multifocal with TTD ≤ 1 cm (n = 211); Group E: PTMC multifocal with TTD >1 cm (n = 179).

### Surgical strategy

During the past few years in our centre, multifocality was often detected in the postsurgical pathology, and local recurrence was commonly found among PTMC patients who had undergone partial thyroidectomy. For these reasons, since 2003, we perform total thyroidectomy plus bilateral central neck dissection for all PTMC patients, regardless of the size, number of foci, and disease stage. When preoperative imaging tests such as ultrasound indicate suspected LNM in the lateral neck, lateral neck dissection is performed. Fine-needle aspiration biopsy and/or intraoperative frozen section examination is routinely performed during thyroid surgical procedures. False-negative cases who underwent lobectomy and in whom more than one microcarcinoma focus was found by routine pathology undergo residual thyroid resection with central lymph node dissection; that is, resection of the thyroid gland and the adjacent nodes bordered superiorly by the hyoid bone, inferiorly by the brachiocephalic vessels, and laterally on each side by the carotid sheaths.

### Pathological confirmation

Routine pathological examination was performed on the whole specimen with serial sectioning at 3-µm intervals for hematoxylin and eosin staining, and the tumours were diagnosed by two experienced pathologists from the Department of Pathology of Union hospital according to the criteria of the World Health Organization. Multifocality was considered when two or more foci were found in one or both lobes; for multifocal PTMC, the largest, dominant tumour was first analysed. Subsequently, the TTD was calculated as the sum of the maximal diameter of each lesion and used for further analysis. Recurrence/disease-persistence was defined as the appearance of pathologically proven malignant tissue and/or the appearance of metastatic lesions in the lungs, bones, and/or brain by imaging studies.

### Statistical Analysis

The patient parameters were compared using Student’s t-test for continuous variables and the Chi-square or Fisher’s exact test for categorical variables. Continuous variables are presented as the mean ± standard deviation or as medians and ranges, and categorical variables are presented as percentages and absolute numbers. Multivariate analysis of all variables was performed using a logistic regression model. Survival outcomes were analysed using the Kaplan-Meier method and log-rank test. All reported p values in this study are 2-sided, and p values < 0.05 were considered statistically significant. All statistical analyses were performed using SPSS software, version 13.0 (SPSS, Chicago, IL).
